# Supplementary material for: β-Phenylethylamine as a Natural Food Additive Shows Antimicrobial Activity against Listeria monocytogenes on Ready-to-Eat Foods
Source: Foods. 2020 Sep 25;9(10):1363. doi: 10.3390/foods9101363 (PMC7600551; doi:10.3390/foods9101363)
Supplement: Supplementary file 1 [file foods-09-01363-s001.pdf]

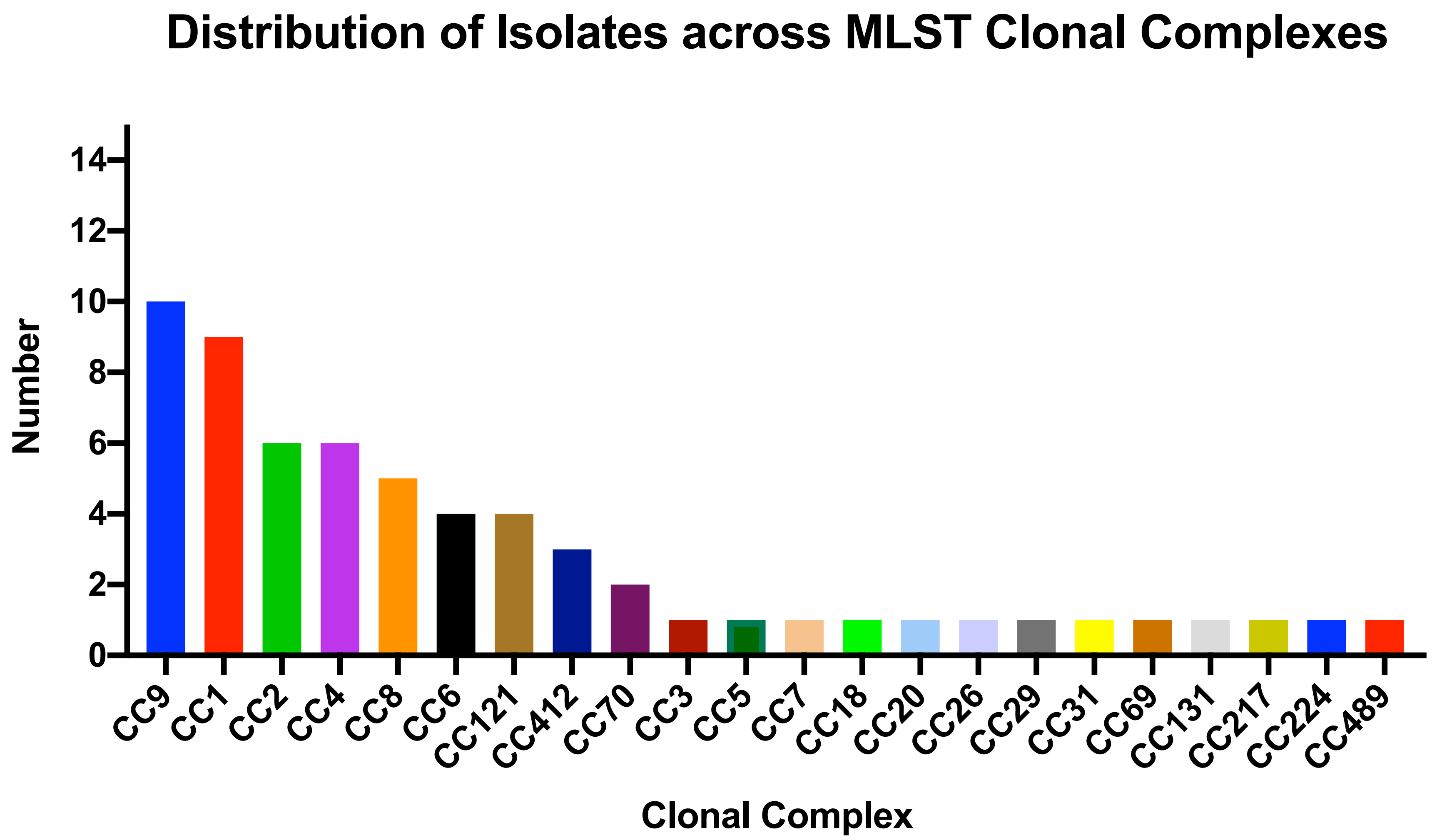

Figure S1: Distribution of isolates across MLST Clonal Complexes. The study strains cover clinical and food relevant *Listeria monocytogenes* genetic backgrounds.

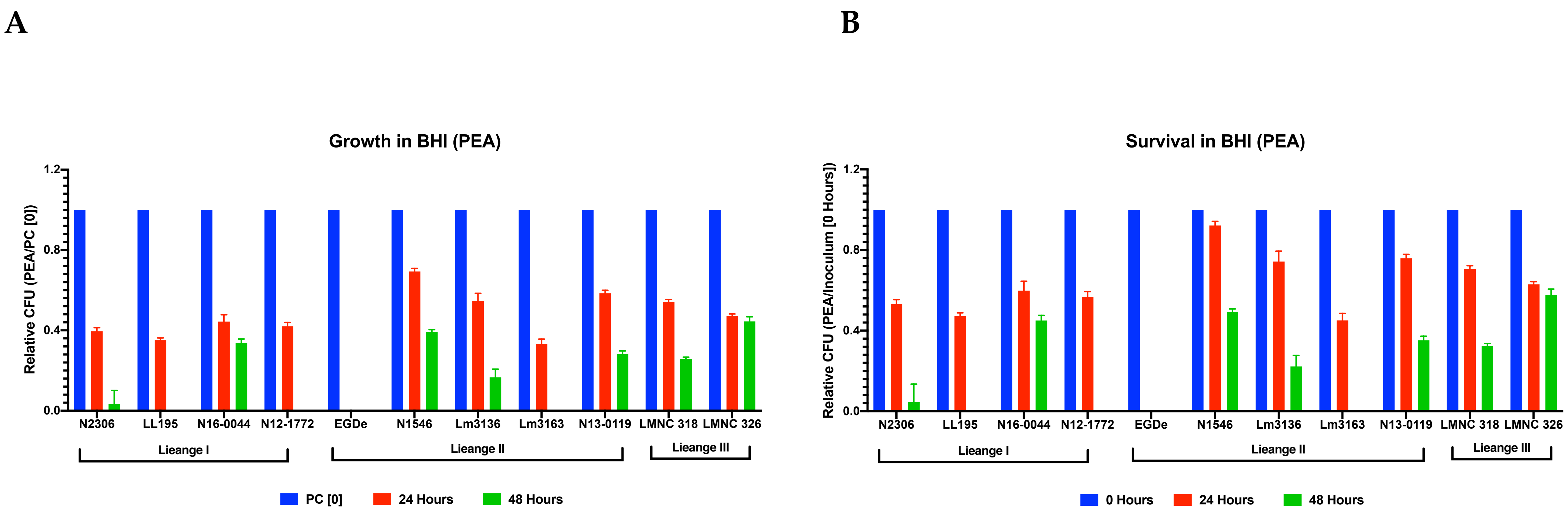

Figure S2: PEA has inhibitory and bactericidal activity against *L. monocytogenes*. Inhibition factors and survival rates of a representative selection of 11 *L. monocytogenes* strains determined after 24 and 48 hours of exposure to PEA MIC in brain heart infusion medium (BHI) at 37 ^{o}C. Presented data showing mean relative colony forming unit (CFU) counts (bars) and standard deviation (error bars) of three independent biological experiments at 0 (start of incubation), 24 and 48 hours of incubation at PEA MIC. **PC [0]** represents the positive control (not treated with PEA but inoculated) CFU counts at each respective time point. For all strains, there was a statistically significant difference between cell counts at each of their respective sampling time points, $P < 0.05$ based on one-way ANOVA and Tukey post-hoc test pairwise comparison of all the treatment conditions.

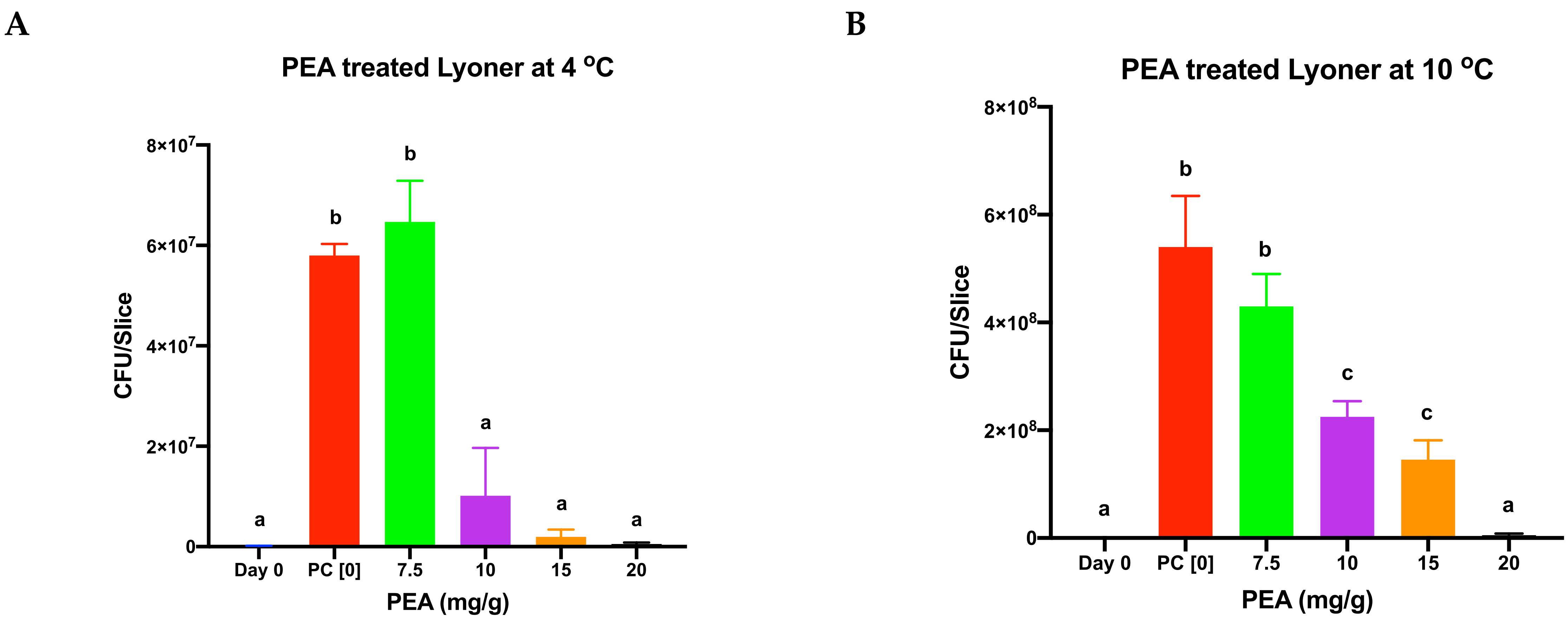

Figure S3: Effect of β-phenylethylamine (PEA) on *L. monocytogenes* growth on lyoner slices treated with different PEA concentrations and inoculated with a cocktail of four *L. monocytogenes* strains. After 11 or 7 days of storage at 4 °C or 10 °C, respectively, CFU per slice from each treatment were determined. Presented data shows the mean CFU per slice (bars) and standard deviation (error bars) of three independent biological experiments. Different letters indicate statistically significant differences between treatments where $P < 0.05$ based on one-way ANOVA and Tukey post-hoc test pairwise comparison of all the treatment conditions.

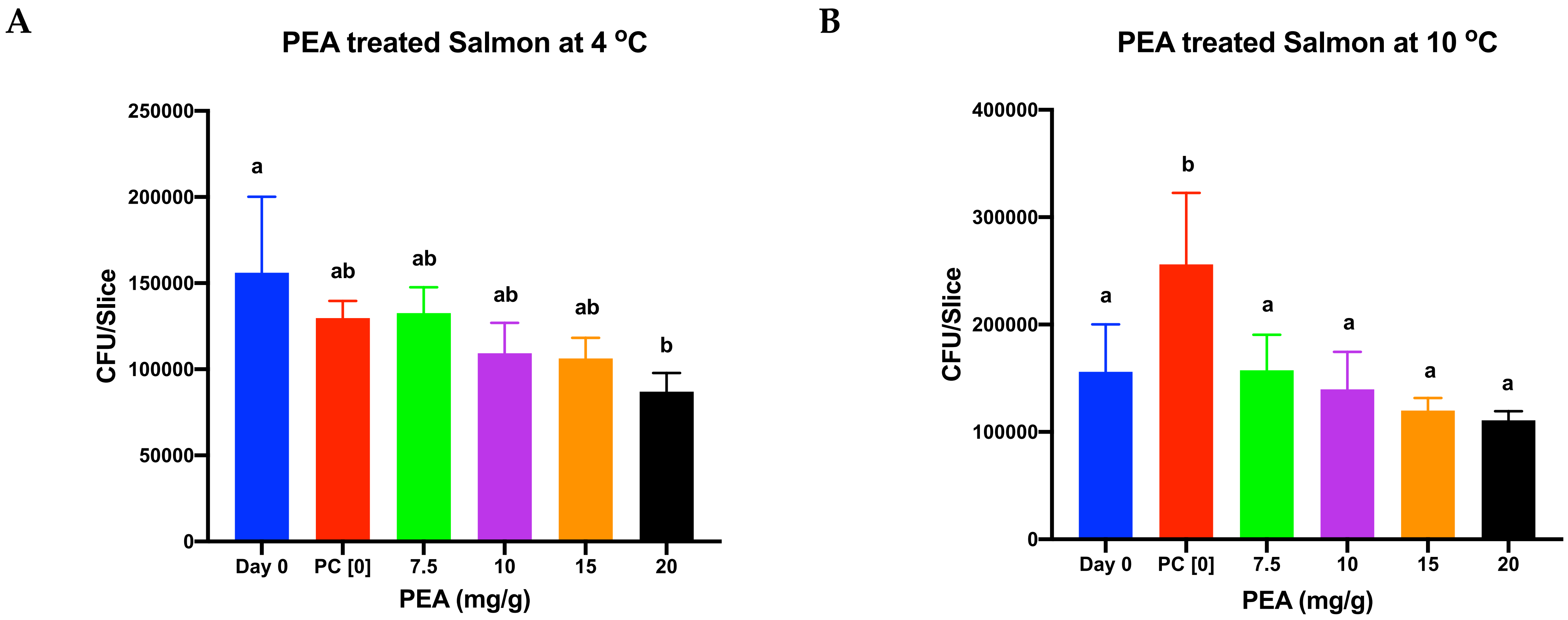

Figure S4: Effect of β-phenylethylamine (PEA) on *L. monocytogenes* growth on smoked salmon slices treated with different PEA concentrations and inoculated with a cocktail of four *L. monocytogenes* strains. After 11 or 7 days of storage at 4 ºC or 10 ºC, respectively, CFU per slice from each treatment were determined. Presented data shows the mean CFU per slice (bars) and standard deviation (error bars) of three independent biological experiments. Different letters indicate statistically significant differences between treatments where $P < 0.05$ based on one-way ANOVA and Tukey post-hoc test pairwise comparison of all the treatment conditions. At 4 ºC significant difference is between the inoculum and 20mg/g PEA treated samples only.

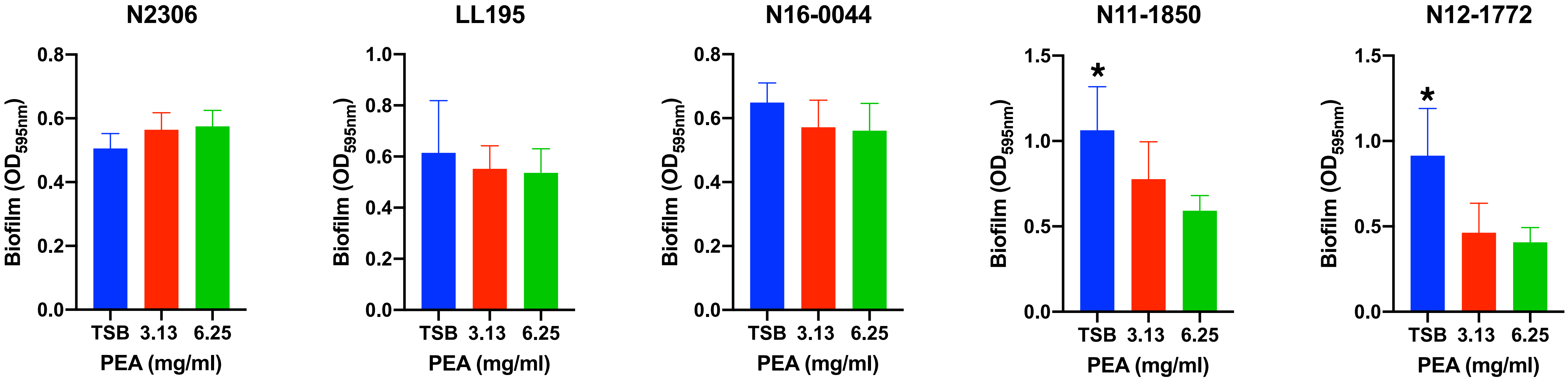

Figure S5A: Effect of β-phenylethylamine (PEA) on biofilm production of lineage I strains. A strain dependent inhibition pattern was observed with the high biofilm producing strains being inhibited in their ability to produce biofilms by PEA at sub-MIC concentrations. Presented data shows the mean biofilm bound bye and standard deviation (error bars) of three independent biological experiments. The asterisk (*) indicates statistically significant differences between treatments where $P < 0.05$ based on one-way ANOVA and Tukey post-hoc test pairwise comparison of all the treatment conditions.

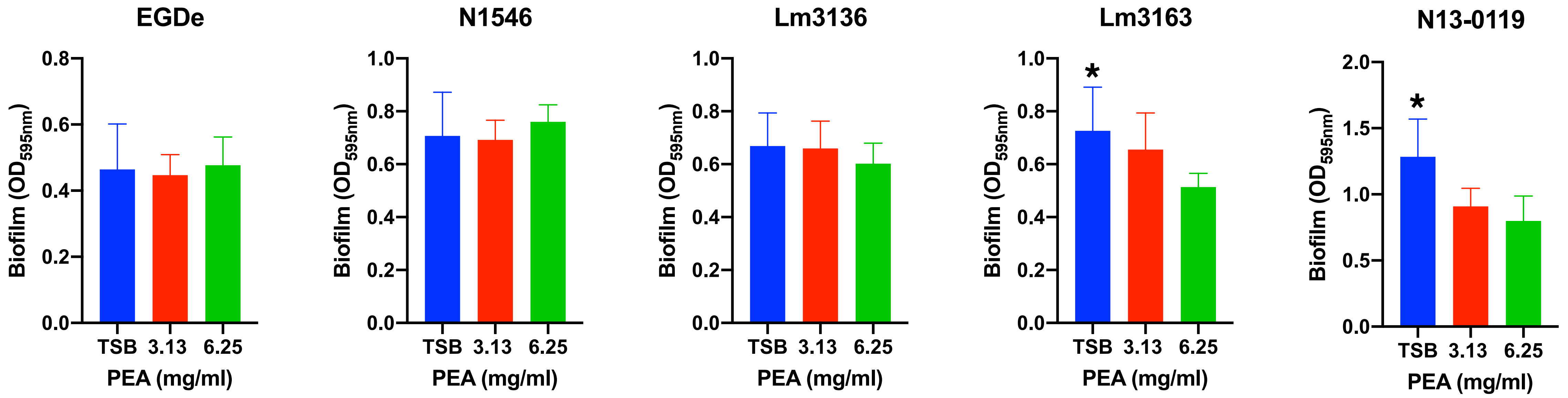

Figure S5B: Effect of β-phenylethylamine (PEA) on biofilm production of lineage II strains. A strain dependent inhibition pattern was observed with the high biofilm producing strains being inhibited most in their ability to produce biofilms by PEA at sub-MIC concentrations. Presented data shows the mean biofilm bound bye and standard deviation (error bars) of three independent biological experiments. The asterisk (*) indicates statistically significant differences between treatments where $P < 0.05$ based on one-way ANOVA and Tukey post-hoc test pairwise comparison of all the treatment conditions.

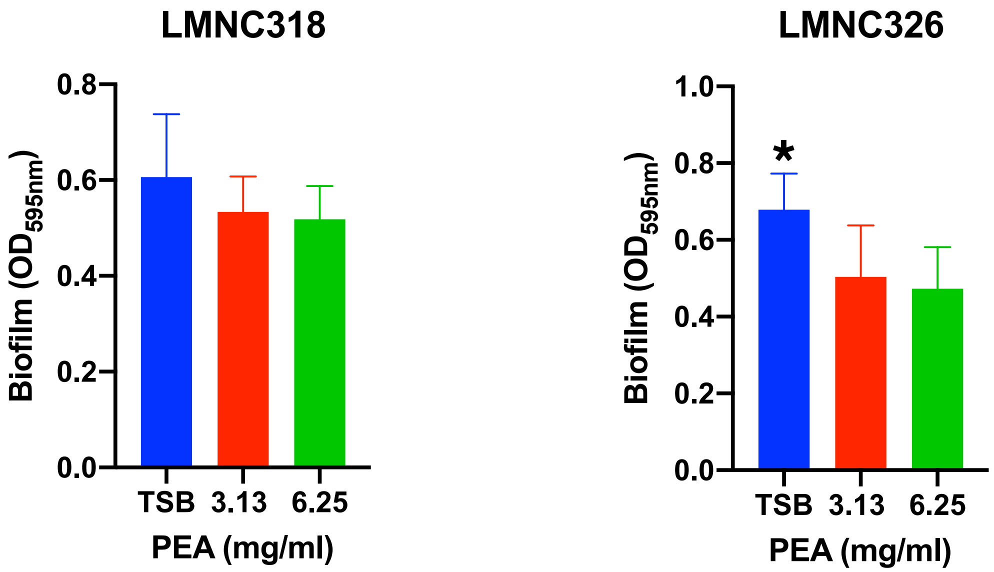

Figure S5C: Effect of β-phenylethylamine (PEA) on biofilm production of lineage III strains. A strain dependent inhibition pattern was observed, sub-MIC concentrations of PEA were used in this assay. Presented data shows the mean biofilm bound bye and standard deviation (error bars) of three independent biological experiments. The asterisk (*) indicates statistically significant differences between treatments where $P < 0.05$ based on one-way ANOVA and Tukey post-hoc test pairwise comparison of all the treatment conditions.

Table S1. *L. monocytogenes* EGDe growth kinetics at different PEA concentrations

	Growth Kinetics		
**BHI PEA (mg/ml)**	**Lag** (hrs)[a]	**Growth Rate**[b]	**AUC**[c]
**0**	3.55 ± 0.06	0.31 ± 0.01	22.25 ± 0.04
**1.56**	3.83 ± 0.10	0.28 ± 0.01	20.83 ± 0.02
**3.13**	4.37 ± 0.11	0.25 ± 0.01	20.06 ± 0.01
**6.25**	9.86 ± 0.10	0.18 ± 0.01	14.38 ± 0.11
**8**	No growth	No growth	No growth

[a]Lag phase duration; [b]Maximum growth rate (OD_{600}/Hour); [c]Area under the curve (AUC).

Table S2. Study strains and their PEA minimum inhibitory concentration (MIC) in different media

Strain distribution and source							Media tested and PEA MIC (mg/ml)				
**Strain ID**	**Source**	**ST**[d]	**CC**[d]	**Serotypes**	**Lineage**	**Reference**	**BHI**	**BHI-A**[c]	**MSM**[c]	**HT BHI**[c]	**HT MSM**[c]
N2306[ab]	Ready-to-eat salads	ST4	CC4	4b	I	Stephan et al., 2015	10	10	10	10	10
LL195[ab]	Vacherin Montd'or cheese	ST1	CC1	4b	I	Bille, 1990	10	10	10	10	10
N14-195	Meat / Meat product	ST31	CC31	4b	I	Ebner et al., 2015	10	10	10	-	-
N16-0044[ab]	Meat pâté	ST6	CC6	4b	I	Althaus et al., 2017	10	10	10	10	10
H34	Human listeriosis	ST489	CC489	1/2b	I	Muchaamba et al., 2018	10	10	10	-	-
N11-2292	human listeriosis	ST1	CC1	4b	I	Althaus et al., 2014	12.5	12.5	12.5	-	-
N11-2675	human listeriosis	ST1063	CC5	1/2b	I	Althaus et al., 2014	10	10	10	-	-
N14-0435	Milk product	ST3	CC3	1/2b	I	Ebner et al., 2015	10	10	10	-	-
N14-0487	Plant associated	ST4	CC4	4b	I	Ebner et al., 2015	10	10	10	-	-
N12-0605	Meat / Meat product	ST727	CC1	4b	I	Ebner et al., 2015	10	10	10	-	-

N12-1339	Meat / Meat product	ST746	CC1	4b	I	Ebner et al., 2015	10	10	10	-	-
N12-1996	Milk / Milk product	ST1	CC1	4b	I	Ebner et al., 2015	10	10	10	-	-
N13-0047	Milk / Milk product	ST1	CC1	4b	I	Ebner et al., 2015	10	10	10	-	-
Scott A	Human listeriosis	ST290	CC2	4b	I	Fleming et al., 1985	10	10	10	-	-
N11-2747	human listeriosis	ST1	CC1	4b	I	Althaus et al., 2014	10	10	10	-	-
N12-0341	human listeriosis	ST1	CC1	4b	I	Althaus et al., 2014	10	10	10	-	-
N12-0551	human listeriosis	ST1	CC1	4b	I	Althaus et al., 2014	10	10	10	-	-
N12-0320	human listeriosis	ST4	CC4	4b	I	Althaus et al., 2014	10	10	10	-	-
N12-0794	human listeriosis	ST4	CC4	4b	I	Althaus et al., 2014	10	10	10	-	-
N13-2107	Meat / Meat product	ST4	CC4	4b	I	Ebner et al., 2015	10	10	10	-	-
N13-1054	human listeriosis	ST1285	CC2	4b	I	Althaus et al., 2014	10	10	10	-	-
N12-1387	human listeriosis	ST6	CC6	4b	I	Althaus et al., 2014	10	10	10	-	-
N11-2801	human listeriosis	ST6	CC6	4b	I	Althaus et al., 2014	10	10	10	-	-
N12-1772[a]	Milk / Milk product	ST682	CC4	4b	I	Ebner et al., 2015	10	10	10	10	10
N13-1184	Meat / Meat product	ST6	CC6	4b	I	Ebner et al., 2015	10	10	10	-	-
N12-0973	Meat / Meat product	ST2	CC2	4b	I	Ebner et al., 2015	10	10	10	-	-
N11-1846	Meat / Meat product	ST724	CC2	4b	I	Ebner et al., 2015	10	10	10	-	-
N12-0432	Meat / Meat product	ST2	CC2	4b	I	Ebner et al., 2015	10	10	10	-	-
N11-1850[a]	Milk / Milk product	ST1290	CC217	4b	I	Ebner *et al*., 2015	10	10	10	10	10
N12-0466	Meat / Meat product	ST2	CC2	4b	I	Ebner *et al*., 2015	10	10	10	-	-
N12-1608	human listeriosis	ST224	CC224	1/2b	I	Althaus *et al*., 2014	10	10	10	-	-
Lm1043S	Human listeriosis	ST85	CC7	1/2a	II	Edman et al., 1968	10	10	10	-	-
N1546[a]	Imported cooked ham	ST8	CC8	1/2a	II	Hächler et al., 2013	10	10	10	10	10
Lm3136[a]	Tomme cheese	ST18	CC18	1/2a	II	Bille et al., 2006	10	10	10	10	10
Lm3163[a]	Tomme cheese	ST26	CC26	1/2a	II	Bille et al., 2006	10	10	10	10	10
N586	Human prosthetic joint	ST412	CC412	3a	II	Muchaamba et al., 2020	10	10	10	-	-

N843	Human prosthetic joint	ST412	CC412	3a	II	Muchaamba et al., 2020	10	10	10	-	-
EGDe[ab]	Rabbits	ST35	CC9	1/2a	II	Glaser et al., 2001	8	8	8	8	8
N11-1515	Milk product	ST29	CC29	1/2a	II	Ebner et al., 2015	10	10	10	-	-
N11-1617	Meat / Meat product	ST8	CC8	1/2a	II	Ebner et al., 2015	10	10	10	-	-
N11-2183	Plant associated	ST20	CC20	1/2a	II	Ebner et al., 2015	10	10	10	-	-
D:824/5	Meat product	ST9	CC9	3c	II	Ebner et al., 2015	10	10	10	-	-
N11-1514	Meat / Meat product	ST9	CC9	1/2c	II	Ebner et al., 2015	10	10	10	-	-
N12-1921	Plant associated	ST9	CC9	1/2c	II	Ebner et al., 2015	10	10	10	-	-
D: 650/8	Meat / Meat product	ST9	CC9	3c	II	Ebner et al., 2015	10	10	10	-	-
N12-0152	Milk / Milk product	ST9	CC9	1/2a	II	Ebner et al., 2015	10	10	10	-	-
N11-1837	human listeriosis	ST9	CC9	1/2a	II	Althaus et al., 2014	10	10	10	-	-
N12-0486	human listeriosis	ST9	CC9	1/2c	II	Althaus et al., 2014	10	10	10	-	-
N13-0001	human listeriosis	ST9	CC9	1/2c	II	Althaus et al., 2014	10	10	10	-	-
N12-1864	Milk / Milk product	ST9	CC9	1/2a	II	Ebner et al., 2015	10	10	10	-	-
N11-1649	Meat / Meat product	ST743	CC8	1/2a	II	Ebner et al., 2015	10	10	10	-	-
N11-1584	human listeriosis	ST1295	CC8	1/2a	II	Althaus et al., 2014	10	10	10	-	-
N12-1273	Human listeriosis	ST412	CC412	1/2a	II	Althaus *et al*., 2014	10	10	10	-	-
N11-1346	Human listeriosis	ST673	CC8	1/2a	II	Althaus *et al*., 2014	12.5	12.5	12.5	-	-
N11-1905	Meat / Meat product	ST121	CC121	1/2a	II	Ebner *et al*., 2015	10	10	10	-	-
N12-1024	Meat / Meat product	ST121	CC121	1/2a	II	Ebner *et al*., 2015	10	10	10	-	-
N13-0119[a]	human listeriosis	ST121	CC121	1/2a	II	Althaus *et al*., 2014	10	10	10	10	10
N12-0367	human listeriosis	ST121	CC121	1/2a	II	Althaus *et al*., 2014	10	10	10	-	-
WSLC1019	Animal isolate	ST130	CC69	4c	III	ATCC 19116	10	10	10	-	-
LMNC318[a]	Ruminant listeriosis	ST70	CC70	4a/4c	III	Oevermann Lab	12.5	12.5	12.5	12.5	12.5
LMNC326[a]	Ruminant listeriosis	ST70	CC70	4a/4c	III	Oevermann Lab	12.5	12.5	12.5	12.5	12.5
WLSC1020	Animal isolate	ST71	CC131	4a	III	ATCC 19114	10	10	10	-	-

[a]Strains used in the biofilm assays as well as assays to evaluate heat stability of PEA activity against *L. monocytogenes*. [b]Strains used in assays to evaluate the effect of PEA on *L. monocytogenes* using food models. [c]Brain heart infusion medium (BHI) agar (BHI-A), meat simulation media (MSM), heat treated BHI broth (HT BHI) and heat treated MSM (HT MSM). [d]ST: sequence type; CC: clonal complex. (-) no test done under that specific condition.

Table S3. Clonal Complex and PEA MICs

	**Strain numbers inhibited at each PEA MIC[a] concentrations**		
**Clonal Complex**	**8mg/ml**	**10mg/ml**	**12mg/ml**
CC1	-	8	1
CC2	-	6	-
CC3	-	1	-
CC4	-	6	-
CC5	-	1	-
CC6	-	4	-
CC7	-	1	-
CC8	-	4	1
CC9	1	9	-
CC18	-	1	-
CC20	-	1	-
CC26	-	1	-
CC29	-	1	-
CC31	-	1	-
CC69	-	1	-
CC70	-	-	2
CC121	-	4	-
CC131	-	1	-
CC217	-	1	-

CC224	-	1	-
CC412	-	3	-
CC489	-	1	-
**Total**	**1**	**57**	**4**

[a]Presented PEA MIC were determined in BHI broth, BHI agar, and MSM.

Table S4. Working solution pH and water activity

BHI PEA (mg/ml)	pH	Water activity
**0**	7.60 ± 0.01	0.993 ± 0.002
**10**	7.60 ± 0.01	0.993 ± 0.001
**15**	7.61 ± 0.02	0.991 ± 0.000
**20**	7.60 ± 0.01	0.991 ± 0.000
**16% NaCl[a]**	7.35 ± 0.00	0.897 ± 0.002

[a]Included as a control for water activity measurement.
